# Contingency Management for Treatment of Cannabis Use Disorder in Co-Occurring Mental Health Disorders: A Systematic Review

**DOI:** 10.3390/brainsci13010036

**Published:** 2022-12-23

**Authors:** Justyne D. Rodas, Maryam Sorkhou, Tony P. George

**Affiliations:** 1 Centre for Complex Interventions and Addictions Division, Centre for Addiction and Mental Health, Toronto, ON M6J 1H4, Canada; 2 Institute of Medical Science, University of Toronto, Toronto, ON M5S 1A8, Canada; 3 Department of Psychiatry, University of Toronto, Toronto, ON M5T 1R8, Canada

**Keywords:** cannabis, contingency management, psychiatric disorders, major depressive disorder, schizophrenia

## Abstract

Amongst individuals with a mental health disorder, a comorbid diagnosis of cannabis use disorder (CUD) is associated with numerous adverse consequences, including more severe symptom profiles, poorer treatment response, and reduced psychosocial functioning. Contingency management (CM), a method to specifically reinforce target behavior attainment (e.g., substance use abstinence), may provide an effective intervention in treating cannabis use in patients with a dual diagnosis of CUD and a mental health disorder. A systematic search examining the effects of CM on cannabis use, clinical, cognitive, and psychosocial outcomes in patients with a mental health disorder on PubMed, PsycINFO, and EMBASE databases up to November 2022 was performed. Six studies met inclusion criteria for our review. We found CM to be efficacious in producing cannabis use reductions and abstinence amongst individuals with a psychotic-spectrum or major depressive disorder. Additional longitudinal studies with larger sample sizes, other psychiatric populations, and longer follow-up periods are needed to evaluate the sustained effects of CM.

## 1. Introduction

Following alcohol and nicotine, cannabis is the most commonly used psychoactive drug, with an estimated 4% of the global population between 15 and 64 using cannabis at least once in 2019 [1]. According to the 3rd edition of the National Epidemiologic Survey on Alcohol and Related Conditions (NESARC-III), 6.3% of adults meet DSM-IV criteria for lifetime cannabis use disorder (CUD) [2]. However, in comparison to the general population, rates of cannabis use and CUD are significantly elevated among those with mental health disorders [3,4]. Using data collected from the NESARC-III, Lev-Ran et al. [5] reported that individuals with a mental health disorder were almost 10 times as likely to use cannabis weekly or suffer from a CUD, compared to individuals without a mental health disorder. Similarly, in a study examining over 15 million adult (18–65 years old) hospitalizations to American acute care community general hospitals between 2007 and 2011, Zhu and Wu [6] found that 62% of the 65,767 inpatients with CUD had a comorbid psychiatric diagnosis, compared to 27.3% of the inpatients without a CUD diagnosis.

Amongst individuals with mental health disorders, a comorbid diagnosis of CUD is clinically relevant because its presence is frequently associated with a poorer prognosis for CUD, the other mental health disorder, or both [7]. Problematic cannabis use in individuals with major depressive disorder (MDD) may lead to clinically significant adverse consequences, including more frequent recurrence of depressive episodes, and poorer treatment adherence [8,9]. Similar findings have been obtained in bipolar disorder and psychotic-spectrum disorders, where cannabis users demonstrate greater symptom severity than nonusers, are at greater risk of hospitalization, and report reduced global and psychosocial functioning [10,11,12,13].

Despite the clear need for effective treatments for problematic cannabis use in individuals with a mental health disorder, no such guidelines have been established. A systematic review of 32 randomized controlled trials examining psychosocial interventions for substance misuse in severe mental health disorders revealed no compelling evidence to support any one psychosocial treatment in reducing either substance use or improving clinical symptom severity among patients [14].

Contingency management (CM) is an operant conditioning-based intervention for substance use disorders (SUDs) that employs rewards, such as money or redeemable vouchers, to reinforce target behaviors, including abstinence from substance use or attendance of treatment sessions. Increasingly, CM is being used within the field of addictions research and has demonstrated promising results. Multiple meta-analyses have demonstrated its effectiveness in reducing alcohol, tobacco, amphetamine, cocaine, cannabis, and opiate use, with medium to large effect sizes [15,16,17]. Studies comparing CM against other psychosocial interventions have typically concluded CM as superior to other behavioral and pharmacological treatments [18,19,20]. Moreover, among individuals with severe mental illness (SMI), CM appears effective in reducing substance use. In a recent meta-analysis examining the effect of CM in patients with a psychotic-spectrum disorder and comorbid SUD, Destoop et al. [21] found CM to offer a small, significant clinical advantage over standard treatment options on abstinence rates. Relative to control conditions involving treatment as usual, patients who received CM in conjunction with standard care were more likely to obtain abstinence post-treatment and at follow-up [21]. However, of the five publications included in this meta-analysis, only one of the studies examined cannabis use. Furthermore, only patients with psychotic disorders were included in this quantitative synthesis. Therefore, this systematic review aims to extend the literature by investigating the efficacy of CM in reducing cannabis use among individuals with a mental health disorder and comorbid CUD. Secondary outcomes included other measures of psychiatric symptoms, psychosocial functioning, and cognition. To our knowledge, this is the first systematic review examining the effectiveness of CM for CUD and comorbid mental health disorders.

## 2. Methods

### 2.1. Search Strategy and Selection Criteria

The review was carried out in accordance with PRISMA guidelines [22]. Searches were performed in November 2022 using PubMed, PsycINFO and EMBASE databases using the following search terms: Schizophrenia OR anxiety OR depression OR posttraumatic stress disorder OR bipolar disorder OR mental health disorder OR severe mental illness AND cannabis OR marijuana AND contingency management. Eligible papers were extracted using the following inclusion criteria: (1) English language articles published in peer-reviewed journals, (2) studies including patients with a mental health disorder as defined by the ICD or DSM, (3) studies including patients who were primarily seeking treatment for cannabis use and (4) studies including at least one arm that administered CM for reducing cannabis use. CM was defined a priori as any intervention that consistently administered rewards (e.g., monetary-, voucher-, or prize-based) to positively reinforce cannabis use reduction or abstinence in patients. Subsequently, the exclusion criteria were: (1) Non-English language papers, (2) Studies not testing CM interventions for cannabis use, (3) Studies including patients without a comorbid mental health disorder, (4) studies administering CM for substances other than cannabis, (5) animal studies, and (6) Other systematic reviews, meta-analyses, abstracts, conference presentations, and case studies. There was no start date or age limitation on the search. The review protocol was not registered or published prior to initiating the study.

Two reviewers (JR and MS) jointly reviewed studies for eligibility by screening titles, abstracts, and subsequently full-text articles. At every stage of the search, all discrepancies were settled by discussion and resolved by mutual consent and/or discussion with the senior author (TPG). Further, reference lists were reviewed among all studies included in the full-text review stage in search for other pertinent publications.

### 2.2. Data Extraction and Risk of Bias

Data were extracted by two reviewers (JR and MS) on populations, study design, classification tool to diagnose CUD and the comorbid mental health disorder, primary and secondary outcomes, and CM reward used. Risk of bias assessments were performed independently by JR, using Cochrane’s Risk-of-Bias tool for non-randomized studies of interventions (RoBINS-I). The ROBINS-I tool assesses the risk of bias across seven prescribed domains, including baseline and time-varying confounding, participant selection, intervention classification, co-intervention, missing data, outcome measurement and selective reporting bias. Following ROBINS-I guidelines [23], a series of signaling questions were considered for each criterion and were rated as having low, moderate, severe, critical, or unclear risk of bias. A study is judged to have an overall low risk of bias when all seven domains are ranked low risk. A study with an overall moderate risk of bias has low or moderate risk across all domains. A study is considered to have a serious risk of bias when at least one domain is at serious risk, but not at critical risk in any domain. Finally, a study is judged to be at critical risk of bias if at least one domain is at critical risk [23]. 

## 3. Results

### 3.1. Study Characteristics

**Figure 1:**
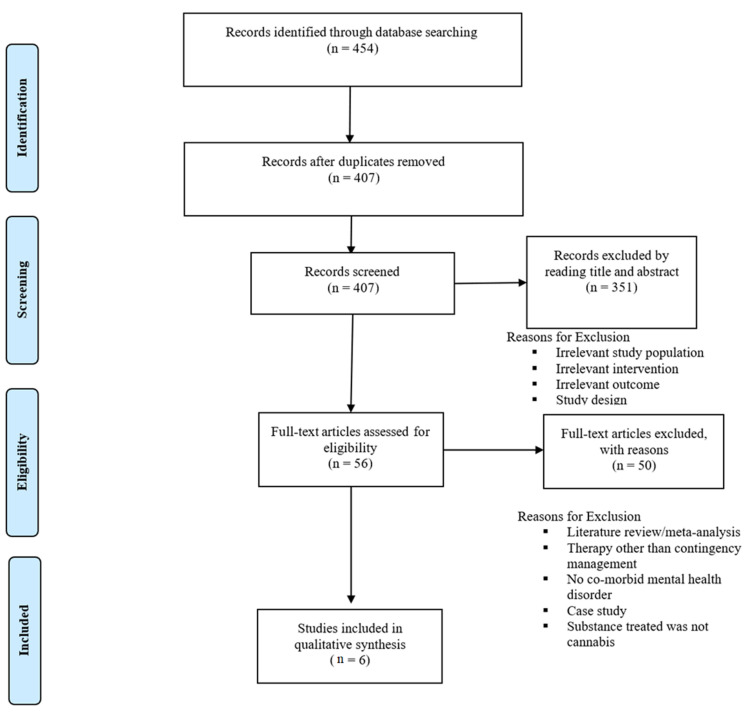
PRISMA Flow Diagram.

Our initial search identified 454 results. Following title and abstract screening, 56 studies were assessed for full-text eligibility. Of these, a total of 6 studies met inclusion criteria and were included in the final review. Specific reasons for exclusion are listed in the PRISMA flowchart (Figure 1).

Characteristics of the 6 included studies for our systematic review are presented in Table 1. The studies were published between 2000 and 2022 and follow a total of 75 participants. Most studies involved patients with a schizophrenia or schizoaffective diagnosis (n = 4), followed by patients with MDD (n = 2). Concerning secondary outcomes, 4 studies examined clinical symptoms, whereas 2 studies examined cognition. None of the studies assessed global or psychosocial functioning as a secondary outcome. Results from the risk of bias assessment are presented in Table 2.

### 3.2. Schizophrenia and Schizoaffective Disorder

Four publications investigated the use of CM in individuals with schizophrenia or schizoaffective disorder [25,26,27,28]. A 25-week cohort study in male cannabis users diagnosed with schizophrenia or supported the use of monetary contingency reinforcement (CR) using escalating contingent payments to reduce cannabis use [27]. Using a within-subject design involving five 5-week conditions, participants were asked to submit urine specimens twice weekly and, depending on the condition, were rewarded a contingent bonus for cannabis-negative results. The authors found that the number of negative urine samples in CR conditions were significantly greater than the baseline conditions. As a secondary aim, changes in psychiatric symptoms across CM and baseline conditions were explored using the Brief Psychiatric Rating Scale (BPRS). However, no differences in symptom severity were observed.

Using a similar within-subject reversal design, Sigmon and Higgins [28] tested the efficacy of voucher-based CR for cannabis abstinence in individuals with serious mental illness. Six individuals with schizophrenia and one with bipolar disorder participated in the 20-week study. The study consisted of three periods: a 4-week baseline, 12-week incentive, and 4-week baseline period, where participants were asked to submit urine specimens twice weekly. During the 12-week incentive condition, participants earned vouchers increasing in value for cannabis-negative urine samples while $10 vouchers were awarded for samples during baseline conditions, independent of toxicology results. Significant increases in the percentage of total and continuous cannabis abstinence were found between baseline conditions and the incentive condition.

A within-subject study explored the effects of cannabis abstinence on cognitive and clinical symptoms in 39 males diagnosed with comorbid schizophrenia and CUD versus non-psychiatric controls [25,26]. Employing a 28-day cannabis abstinence paradigm consisting of monetary CR and motivational interviewing, 42% of individuals with schizophrenia and 55% of controls achieved 28 days of cannabis abstinence. Moreover, individuals with schizophrenia who successfully achieved abstinence demonstrated significant improvements in verbal memory and learning post-treatment [25]. In contrast, non-abstainers with schizophrenia and individuals without a mental health disorder who obtained abstinence did not significantly improve in these outcomes [25]. Concerning clinical outcomes, Rabin et al. [26] did not detect any significant improvements in positive or negative symptoms of schizophrenia in patients obtaining Day 28 abstinence. However, depressive symptoms improved among abstainers in both the experimental and control groups.

### 3.3. Major Depressive Disorder (MDD)

A recent open-label, single-arm study using monetary CM evaluated the cognitive and clinical effects of 28 days of cannabis abstinence among individuals with co-morbid MDD and CUD [24,29]. Upon biochemically confirmed abstinence at Day 28 using THC urine toxicology and self-report, participants were eligible to receive a $300 contingent bonus. Of eleven participants, eight obtained biochemically-verified cannabis abstinence while the remaining participants significantly reduced their cannabis use. Coinciding with abstinence and reductions in use, significant improvements in select cognitive domains, including visual search speed, visual sustained attention and response inhibition, and VSWM were found [29]. Concerning clinical outcomes associated with cannabis abstinence, improvements in depressive symptomology and anhedonia were observed; cannabis abstinence led to non-significant improvements in anxiety symptomology [24]. 

## 4. Discussion

This systematic review examined CM as a method to reduce cannabis use and probe clinical and cognitive symptoms in individuals with comorbid cannabis use and mental health disorders. The findings of our review suggest that CM can be used to produce short- and long-term cannabis abstinence in individuals with schizophrenia, MDD, and other serious mental illnesses. We also highlight the use of CM as a successful methodological tool to examine the effects of cannabis abstinence on several cognitive, substance use, and psychiatric outcomes [24,25,26,27,28,29]; see Table 1.

With many patients achieving cannabis abstinence during the CM periods within the included studies, we provide strong evidence to support the efficacy of CM to promote cannabis abstinence in dually-diagnosed patients, with improvements in clinical symptomatology and some aspects of cognitive dysfunction in these disorders. These results are consistent with previous literature delivering CM to individuals with comorbid SUDs and mental health disorders [17,30,31,32,33,34,35]. While several lines of research suggest that cannabis use is associated with poorer clinical outcomes [7,36], we found that cannabis abstinence did not correspond with positive clinical changes among all psychiatric outcomes. These findings parallel studies employing a randomized, controlled trial using CM to achieve cannabis abstinence in adolescents with subclinical symptoms of depression and anxiety [37,38,39]. In this trial, cannabis-using adolescents who maintained four weeks of abstinence demonstrated significant improvements in verbal learning and memory [39], while only adolescents who were heavy cannabis users obtained small improvements in anxious and depressive symptoms [37]. Nonetheless, our results are encouraging, as many of the included studies did observe a positive effect of cannabis abstinence on depressive symptoms, in addition to cognitive outcomes in both individuals with major depression and schizophrenia [24,25,26,29].

## 5. Strengths and Limitations

One of the major strengths of this study is its focus on clinical outcomes, including cognition, substance use and mental health symptoms. By extracting these variables, we provided evidence to support the use of CM to achieve cannabis abstinence and better understand clinical outcomes of abstinence. These findings may be of significant interest to healthcare providers, researchers, and the general public. Moreover, we employed a methodologically rigorous and comprehensive approach to our review by adhering to PRISMA guidelines.

Although we performed a comprehensive, systematic review, there are several limitations to note. First, our sample size was small due to the limited number of studies exploring the efficacy of CM for CUD in psychiatric populations. Future research with larger sample sizes is required to provide more generalizable findings. Second, most studies did not include a control (non-abstinence) group, making it difficult to assess the specificity of CM’s efficacy on clinical and cognitive outcome measures. Third, a large portion of the studies were conducted in males. Excluding the study examining 28 days of cannabis abstinence in individuals with MDD [24,29], 86–100% of the samples were male, limiting conclusions on the efficacy of CM in substance use, clinical, and cognitive outcomes among females. Fourth, over half of the studies used CM in conjunction with supportive therapy. This raises some concerns about whether the skills acquired from therapy contributed to changes in clinical outcomes; however, studies have shown that brief (e.g., 15–30 min), low-intensity behavioral interventions have minimal effects and closely resembles “treatment as usual” [40]. Fifth, few studies assessed substance substitution. Many cannabis users are polysubstance users, and it is unknown whether abstinence led to compensatory increases in concurrent substance use. There is some evidence to suggest that reductions in cannabis use may be compensated by increased alcohol and tobacco consumption [41,42,43]. Additionally, few studies incorporated secondary outcome measures, and it remains unknown whether cannabis abstinence is associated with improvements in functional outcomes, including social and role functioning. Finally, due to the limited number of studies included within this review, it remains unknown whether CM for cannabis use is efficacious among other psychiatric populations, including individuals with anxiety disorders, posttraumatic stress disorder, and bipolar disorder.

Despite these limitations, our findings may have important clinical implications given that problematic cannabis use disproportionately affects individuals with comorbid mental health disorders [4,36]. Similar to other reviews and meta-analyses [16,21], CM appears as an appropriate treatment for CUD in mental health disorders. 

## 6. Conclusions and Future Directions

In this systematic review, we evaluated published evidence on the use of CM in patients with comorbid cannabis use and mental health disorders. Our findings suggest that CM is a reliable method to increase our understanding of the longitudinal, state-specific effects of cannabis use through abstinence-based procedures. Future research using randomized, controlled designs, with a larger sample size and longer abstinence periods should explore the effects of prolonged abstinence on clinical, cognitive, and psychosocial outcomes in diverse psychiatric populations. Finally, there is a need to incorporate follow-up visits, as it remains unknown whether CM leads to sustained abstinence in individuals with CUD and comorbid mental health disorders.

## Figures and Tables

**Table 1:** Review of Evidence Concerning the Efficacy of Contingency Management on Substance Use and Psychiatric Outcomes.

Reference	Aim	Study Design	Type of Contingency Management	Sample Characteristics (% Male)	Scale Used for SUD Diagnosis	SUD Outcome	SUD Findings	Scale Used for Psychiatric Diagnosis	Mental Health Outcome	Symptom Findings
Lucatch et al., 2020 [24]	To understand the effects of cannabis abstinence on clinical symptoms of depression in individuals with CUD and MDD	Within subject, feasibility, successive cohort	Monetary	N = 11 (27.3%M)	DSM-5	THC-COOH levels; urinalysis, MWC	Cr-THC-COOH levels significantly declined over the 28-day abstinence period across all participants, statistically significant changes in withdrawal severity over time; post-hoc test revealed a significant increase in withdrawal symptoms between weeks 1 and 2	DSM-5	BAI, HAM-D, SHAPS	Reductions in THC were significantly associated with reductions in depression and anhedonia scores over time, while a non-significant trend was determined for reductions in anxiety scores
Sigmon et al., 2000 [25]	To examine the sensitivity of cannabis use to monetary incentives among individuals with schizophrenia and other serious mental illness	Within subject, crossover, feasibility, successive cohort	Monetary	N = 18 (100%M)	DSM-III-R	THC-COOH levels; urinalysis	The average total number and consecutive number of marijuana-negative specimens obtained were greater during the conditions wherein participants received monetary reinforcement contingent on abstinence than in the baseline conditions	DSM-IV	BPRS	No significant changes in psychiatric symptom severity in either baseline or incentive conditions
Sigmon & Higgins, 2006 [26]	To determine the efficacy of voucher-based contingency management in reducing marijuana use in individuals with schizophrenia, schizoaffective disorder, or other serious mental illnesses	Within-subject, reversal design	Voucher	N = 7 (86%M)	DSM-IV	Urinalysis using Abuscreen ONTRAK	Compared to the other conditions, the percentage of negative urine tests were significantly greater during the voucher intervention	DSM-IV	N/A	N/A
Rabin et al., 2017 [27]	To determine the effects of cannabis abstinence on cognition in patients with schizophrenia and co-occurring cannabis dependence	Within subject, feasibility, successive cohort	Monetary	N = 39 (100%M)	DSM-IV-TR	THC-COOH levels; urinalysis	Participant abstinence rates were not significantly different between patients with schizophrenia and non-psychiatric control groups: 42.1% of patients (8/19) and 55% of controls (11/20) successfully achieved abstinence verification criteria	DSM-IV-TR	HVLT, SDR, Digit Span Forwards and Backwards, CPT-II, TMT, grooved pegboard, BART, KDDT, SARS, BARS, AIMS	Patients with schizophrenia who successfully abstained demonstrated improvements in verbal memory and learning; however, findings were insignificant when correcting for multiple comparisons No changes in SARS, BARS, or AIMS scores between baseline and day 28 in patient abstainers and non-abstainers
Rabin et al., 2018 [28]	To determine the effects of cannabis abstinence on clinical symptoms in patients with schizophrenia and co-occurring cannabis dependence	Within subject, feasibility, successive cohort	Monetary	N = 39 (100%M)	DSM-IV-TR	THC-COOH levels; urinalysis, TLFB	Abstaining and relapsing patients and controls, demonstrated a significant decrease in self-reported cannabis consumption over the 28-day study period (patient and control abstainers, *p* < 0.001; and patient and control relapsers, *p* < 0.01)	DSM-IV-TR	PANSS, CDSS, HAM-D	PANSS scores remained constant across the abstinence period in both abstaining and non-abstaining patients Significant main effect of time on CDSS scores between abstainers and non-abstainers; insignificant abstinence status x time interaction No significant effect of time on HAM-D scores
Sorkhou et al., 2022 [29]	To determine whether a 28-day period of cannabis abstinence is associated with improvements in cognition in patients with MDD and comorbid CUD	Within subject, feasibility, successive cohort	Monetary	N = 11 (27.3%M)	DSM-5	THC-COOH levels; urinalysis	Cr-THC-COOH levels significantly declined over the 28-day abstinence period across all participants. Moreover, 8/11 (72.7%) participants met pre-specified criteria for 28 days of cannabis abstinence. In the three participants who failed to meet abstinence (lapsers), Cr-THC-COOH levels decreased substantially (~93%) from Day 0 to Day 28	DSM-5	HVLT, CPT, TMT-A, TMT-B, SDR-30, DS-Forwards, DS-Backwards	Visual search speed, selective attention, and VSWM improved over the study period; improvements were not associated with changes in cannabis metabolite levels from baseline to endpoint

AIMS: Abnormal Involuntary Movement Scale; BAI: Beck’s Anxiety Inventory; BARS: Barnes Akathisia Rating Scale; BART: Balloon Analog Risk Task; BPRS: Brief Psychiatric Rating Scale; CDSS: Calgary Depression Scale for Schizophrenia; CPT: Continuous Performance Test; CUD: Cannabis Use Disorder; DS: Digit Span; HAM-D: Hamilton Rating Scale for Depression; HVLT: Hopkins Verbal Learning Test; KDTT: Kirby Delayed Discounting Test; MDD: Major Depressive Disorder; PANSS: Positive and Negative; SARS: Simpson Angus Rating Scale; SDR: Spatial Delayed Response; SHAPS: Snaith-Hamilton Pleasure Scale; SUD: Substance Use Disorder; TMT: Trail Making Test.

**Table 2:** Risk of Bias Using Cochrane’s Risk of Bias In Non-randomized Studies—of Interventions (ROBINS-I) Tool.

Reference	Bias Due to Confounding	Bias in Selection of Participants into the Study	Bias in Classification of Interventions	Bias Due to Deviations from Intended Interventions	Bias Due to Missing Data	Bias in Measurement of Outcomes	Bias in Selection of the Reported Result	Overall RoB
Lucatch et al., 2020 [24]	Critical	Low	Serious	Low	Serious	Serious	Low	Critical
Rabin et al., 2017 [27]	Moderate	Low	Serious	Low	Low	Serious	Low	Serious
Rabin et al., 2018 [28]	Moderate	Low	Serious	Low	Low	Serious	Low	Serious
Sigmon et al., 2000 [25]	Moderate	Low	Low	Low	Serious	Serious	Moderate	Serious
Sigmon & Higgins, 2006 [26]	Moderate	Low	Low	Low	Moderate	Serious	Low	Serious
Sorkhou et al., 2022 [29]	Critical	Low	Serious	Low	Serious	Serious	Low	Critical

## Data Availability

Not applicable.
